# Differential progression of coronary atherosclerosis according to plaque composition: a cluster analysis of PARADIGM registry data

**DOI:** 10.1038/s41598-021-96616-w

**Published:** 2021-08-24

**Authors:** Yeonyee E. Yoon, Lohendran Baskaran, Benjamin C. Lee, Mohit Kumar Pandey, Benjamin Goebel, Sang-Eun Lee, Ji Min Sung, Daniele Andreini, Mouaz H. Al-Mallah, Matthew J. Budoff, Filippo Cademartiri, Kavitha Chinnaiyan, Jung Hyun Choi, Eun Ju Chun, Edoardo Conte, Ilan Gottlieb, Martin Hadamitzky, Yong Jin Kim, Byoung Kwon Lee, Jonathon A. Leipsic, Erica Maffei, Hugo Marques, Pedro de Araújo Gonçalves, Gianluca Pontone, Sanghoon Shin, Jagat Narula, Jeroen J. Bax, Fay Yu-Huei Lin, Leslee Shaw, Hyuk-Jae Chang

**Affiliations:** 1 Department of Radiology, Dalio Institute of Cardiovascular Imaging, New York-Presbyterian Hospital and Weill Cornell Medicine, New York, NY USA; 2 Department of Internal Medicine, Seoul National University College of Medicine, Cardiovascular Center, Seoul National University Hospital, Seoul, South Korea; 3 Cardiovascular Center, Seoul National University Bundang Hospital, Sungnam, South Korea; 4 Department of Cardiovascular Medicine, National Heart Centre, Singapore, Singapore; 5 Ipsos US Public Affairs, New York, NY USA; 6 Division of Cardiology, Department of Internal Medicine, Ewha Womans University Seoul Hospital, Ewha Womans University College of Medicine, Seoul, South Korea; 7 Yonsei-Cedars-Sinai Integrative Cardiovascular Imaging Research Center, Yonsei University College of Medicine, Yonsei University Health System, Seoul, South Korea; 8 Division of Cardiology, Severance Cardiovascular Hospital, Yonsei University College of Medicine, Yonsei University Health System, Seoul, South Korea; 9 Centro Cardiologico Monzino, Istituto di Ricovero e Cura a Carattere Scientifico (IRCCS), Milan, Italy; 10 Houston Methodist DeBakey Heart and Vascular Center, Houston Methodist Hospital, Houston, TX USA; 11 Department of Medicine, Lundquist Institute at Harbor UCLA Medical Center, Torrance, CA USA; 12 Cardiovascular Imaging Unit, SDN IRCCS, Naples, Italy; 13 Department of Cardiology, William Beaumont Hospital, Royal Oak, MI USA; 14 Pusan University Hospital, Busan, South Korea; 15 Department of Radiology, Casa de Saude São Jose, Rio de Janeiro, Brazil; 16 Department of Radiology and Nuclear Medicine, German Heart Centre Munich, Munich, Germany; 17 Gangnam Severance Hospital, Yonsei University College of Medicine, Seoul, South Korea; 18 Department of Medicine and Radiology, University of British Columbia, Vancouver, BC Canada; 19 Department of Radiology, Area Vasta 1/Azienda Sanitaria Unica Regionale (ASUR) Marche, Urbino, Italy; 20 Unit of Cardiovascular Imaging, UNICA, Hospital da Luz, Lisbon, Portugal; 21 NOVA Medical School, Lisbon, Portugal; 22 Icahn School of Medicine at Mount Sinai, Mount Sinai Heart, Zena and Michael A. Wiener Cardiovascular Institute, and Marie-Josée and Henry R. Kravis Center for Cardiovascular Health, New York, NY USA; 23 Department of Cardiology, Leiden University Medical Centre, Leiden, The Netherlands

**Keywords:** Coronary artery disease and stable angina, Computed tomography

## Abstract

Patient-specific phenotyping of coronary atherosclerosis would facilitate personalized risk assessment and preventive treatment. We explored whether unsupervised cluster analysis can categorize patients with coronary atherosclerosis according to their plaque composition, and determined how these differing plaque composition profiles impact plaque progression. Patients with coronary atherosclerotic plaque (n = 947; median age, 62 years; 59% male) were enrolled from a prospective multi-national registry of consecutive patients who underwent serial coronary computed tomography angiography (median inter-scan duration, 3.3 years). *K*-means clustering applied to the percent volume of each plaque component and identified 4 clusters of patients with distinct plaque composition. Cluster 1 (n = 52), which comprised mainly fibro-fatty plaque with a significant necrotic core (median, 55.7% and 16.0% of the total plaque volume, respectively), showed the least total plaque volume (PV) progression (+ 23.3 mm^3^), with necrotic core and fibro-fatty PV regression (− 5.7 mm^3^ and − 5.6 mm^3^, respectively). Cluster 2 (n = 219), which contained largely fibro-fatty (39.2%) and fibrous plaque (46.8%), showed fibro-fatty PV regression (− 2.4 mm^3^). Cluster 3 (n = 376), which comprised mostly fibrous (62.7%) and calcified plaque (23.6%), showed increasingly prominent calcified PV progression (+ 21.4 mm^3^). Cluster 4 (n = 300), which comprised mostly calcified plaque (58.7%), demonstrated the greatest total PV increase (+ 50.7mm^3^), predominantly increasing in calcified PV (+ 35.9 mm^3^). Multivariable analysis showed higher risk for plaque progression in Clusters 3 and 4, and higher risk for adverse cardiac events in Clusters 2, 3, and 4 compared to that in Cluster 1. Unsupervised clustering algorithms may uniquely characterize patient phenotypes with varied atherosclerotic plaque profiles, yielding distinct patterns of progressive disease and outcome.

## Introduction

Understanding the process of coronary atherosclerosis could facilitate timely medical intervention to retard the development of clinically significant coronary artery disease and its consequences. Coronary computed tomographic angiography (CCTA) which provides noninvasive and comprehensive evaluation of coronary atherosclerosis has been utilized to understand the pathophysiologic progression of coronary atherosclerosis over time^1–3^. Furthermore, CCTA has the potential to personalize preventive therapy by quantitatively evaluating heterogeneous coronary atherosclerotic plaque volume and composition in whole coronary trees^4,5^. However, plaque components form a pathophysiologic continuum, thus it is difficult to determine the threshold of plaque composition in terms of clinical implications.

Machine learning using unsupervised cluster analysis aims to group similar data points into clusters based on inherent similarities among them. It thus enables the exploration of possible heterogeneity within a disease category that has historically been considered homogeneous^6,7^. In the present study, we hypothesized that unsupervised cluster analysis could categorize heterogeneous patients according to atherosclerotic plaque component proportions. Furthermore, we aimed to determine how these differences in atherosclerotic plaque components at baseline differentially impact plaque progression and composition change.

## Methods

### Study design and population

The Progression of AtheRosclerotic PlAque DetermIned by Computed TomoGraphic Angiography Imaging (PARADIGM) study was a multinational observational registry that prospectively enrolled 2252 patients who underwent clinically indicated serial CCTAs at an inter-scan interval of ≥ 2 years at 13 sites in 7 countries between 2003 and 2015^8^. The study protocol complied with the Declaration of Helsinki and was approved by the institutional review boards of all participating centers (Severance Hospital, Gangnam Severance Hospital, Seoul National University Hospital, Seoul National University Bundang Hospital, National Health Insurance Service Ilsan Hospital, and Busan University Hospital, Korea; Weil Cornell Medical College and NewYork-Presbyterian Hospital, and Harbor UCLA Medical Center, USA; St. Paul’s Hospital, Canada; University Hospital of Parma, and IRCCS, Italy; Hospital da Luz, Portugal; University of Munich, Germany; Casa de Saude Sao Jose, Brazil). All the study participants gave informed consent.

For the present analysis, we excluded patients with uninterpretable CCTAs (n = 492), prior revascularization (n = 282), and no coronary atherosclerotic plaque at baseline (n = 358). (Supplementary Fig. 1) To explore the natural history of coronary atherosclerosis, we defined statin-naïve patients as patients who were not using statin at the time of the baseline and follow-up CCTAs. Statin-taking patients were defined as those who were using statin at the time of follow-up CCTA^2^. After further excluding patients without information on statin use (n = 121) and those who discontinued statin use after the baseline CCTA (n = 52), 947 patients remained for the final analysis.

### CCTA analysis

Acquisition and analysis of CCTAs were performed in accordance with guidelines^8^. Data from each participating site were transferred to a core laboratory for blinded image analysis by level-III experienced readers using semi-automated plaque analysis software (QAngioCT Systems, Leiden, the Netherlands) with manual correction^8^.

All coronary artery segments with a diameter ≥ 2 mm were evaluated for plaque and vessel volume (mm^3^) using a modified 17-segment American Heart Association model^9,10^. Segments were matched between baseline and follow-up CCTAs using branch points as landmarks. The presence of atherosclerosis was defined as any tissue ≥ 1 mm^2^ within or adjacent to the lumen that could be discriminated from surrounding pericardial tissue, epicardial fat, or lumen, and identified in ≥ 2 planes. Plaque volume (PV) (mm^3^) was measured and further sub-classified by the composition using pre-defined Hounsfield unit (HU) cut-off values (necrotic core, − 30 to 30 HU; fibro-fatty plaque, 30 to 130 HU; fibrous plaque, 131 to 350 HU; and calcified plaque, ≥ 351 HU)^11,12^. To account for differences in the total vessel length between patients and to provide an equal weighting of each patient in the calculation of PV, we normalized PV as [(absolute PV/the total vessel length) * mean population vessel length]^13^. We calculated the annualized total PV change as (Δtotal PV/CCTA interval, mm^3^/year), and used the median value as a cut-off point to determine the plaque progression.

### Unsupervised clustering

Clustering is an unsupervised technique used to group objects that are “close” to one another in a multi-dimensional feature space, usually to uncover some inherent structure within the data without prior assumptions^7,14^. *K*-means clustering is a vector quantization method used for partitioning *n* observations into a pre-defined number (*k*) of mutually exclusive clusters, in which each observation belongs to the cluster with the nearest mean. The algorithm iteratively minimizes the sum of the square distances between cluster points and the cluster mean. As such, it locally optimizes the following objective function, using an iterative procedure similar to the expectation–maximization algorithm as below (n = the number of data points, K = the pre-defined number of clusters, ${w}_{ik}$= 1 if ${x}^{i}$ belongs to cluster k or 0 otherwise, and ${x}^{i}$= the mean of cluster k)^6^.

$$J={\sum }_{i=1}^{n}{\sum }_{k=1}^{K}{w}_{ik}||{x}^{i}-{\mu }_{k}{||}^{2}$$

To categorize heterogeneous patients according to plaque component proportions, we applied *k*-means clustering to the baseline percent volume (%vol) of each plaque component (necrotic core, fibro-fatty plaque, fibrous plaque, and calcified plaque), defined as (component plaque volume/total plaque volume × 100, %). Clustering was performed independently from clinical and follow-up CCTA data. The number of clusters was selected as 4, based on the Calinski-Harabasz Index and Average Silhouette Width, as well as the elbow method (Supplementary Fig. 2)^6,15^. Since the clustering algorithm does not provide a specific cluster order, we ordered the clusters based on the %vol of calcified plaque.

Clustering was validated with nonparametric bootstrapping (Supplementary Table 1)^16^, and visualized using several techniques including 3-dimensional (3D) plots displaying 3 of 4 features at a time, and the remapping of multi-dimensional plots into 2-dimensional (2D) plots using radial visualization (RadViz) and t-distributed stochastic neighbour embedding (t-SNE)^17,18^.

### Study outcomes

After clustering, we compared the individual clinical phenotypes and changes in PV and characteristics among clusters, followed by an interpretation of the clinical relevance. In 808 patients (85%) with available clinical outcome data, we also compared the composite of major adverse cardiac events (MACE), including all-cause mortality, acute coronary syndrome, and coronary revascularization.

### Statistical analysis

Continuous variables are presented as the median [interquartile range (IQR)]; categorical variables are presented as numbers (percentages). Differences among clusters were evaluated using the analysis of variance or Kruskal–Wallis test for continuous variables, and the χ2 test or Fisher’s exact test for categorical variables, as appropriate, followed by Bonferroni’s correction or Dunn’s post-hoc testing for multiple comparisons. Multivariable logistic regression analysis, including the 10-year atherosclerotic cardiovascular disease (ASCVD) risk, diabetes mellitus, baseline total PV, and statin use, was performed to compare the risk for plaque progression among clusters. Additionally, multivariable Cox regression analysis, including the 10-year ASCVD risk, diabetes mellitus, baseline total PV, annualized total PV change, and statin use, was performed to evaluate the relative hazard for MACE among clusters. Cluster 1 was used as the reference group for multivariable analyses, and the results are expressed as the adjusted odds ratio (aOR) or adjusted hazard ratio (aHR) with the corresponding 95% confidence interval (CI). MACE-free survival data were plotted using the Kaplan–Meier method and compared by the log-rank test. All statistical analyses, including clustering, were performed using RStudio (Version 3.6.3) and its packages. *P* < 0.05 was considered statistically significant.

## Results

### Clustering of patients with coronary atherosclerotic plaque

**Figure 1:**
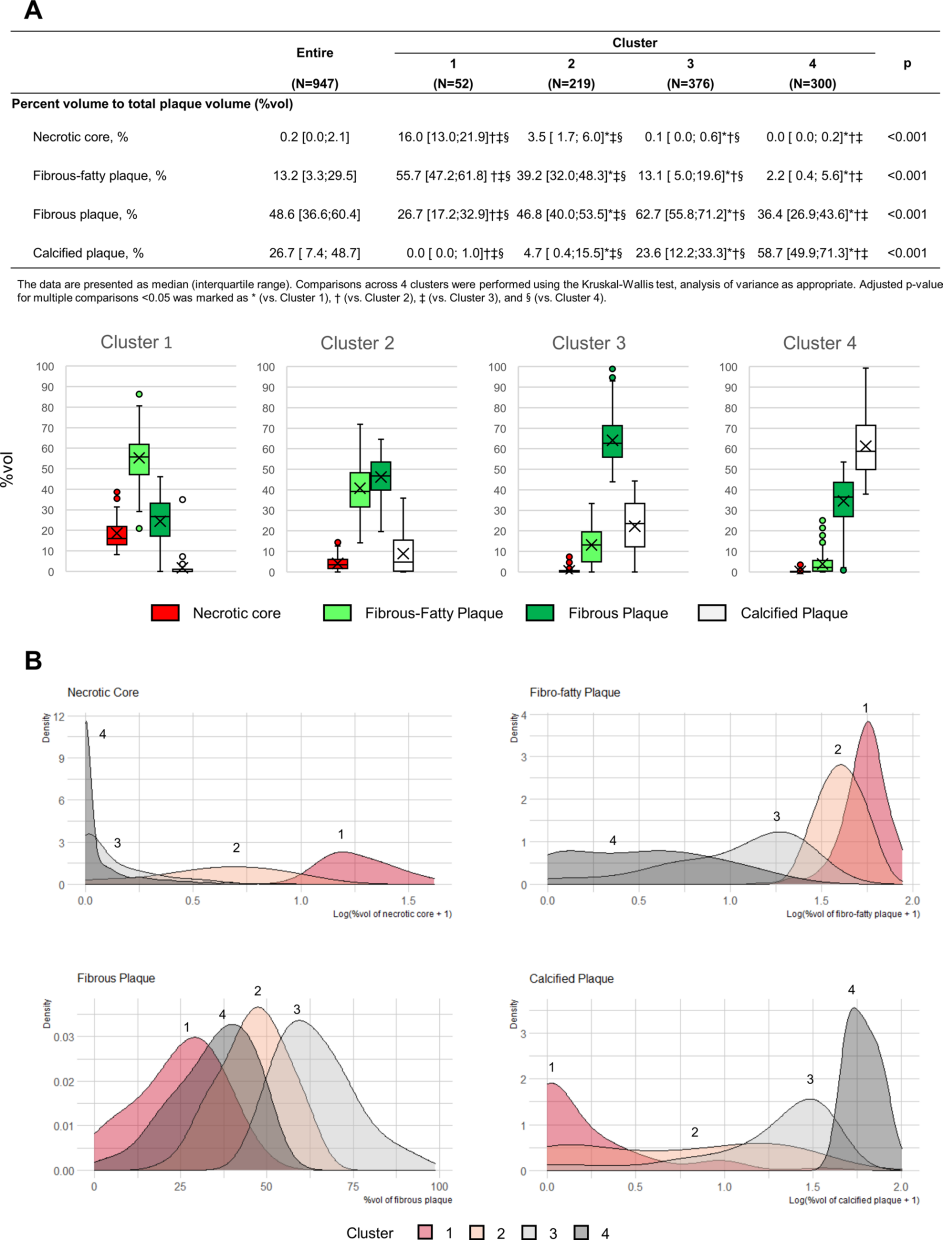
Plaque composition across clusters. (**A**) *K*-means clustering identified 4 groups of patients with different plaque composition. (**B**) Density plots showing the distribution of the percent volume (%vol) of each plaque component.

**Figure 2:**
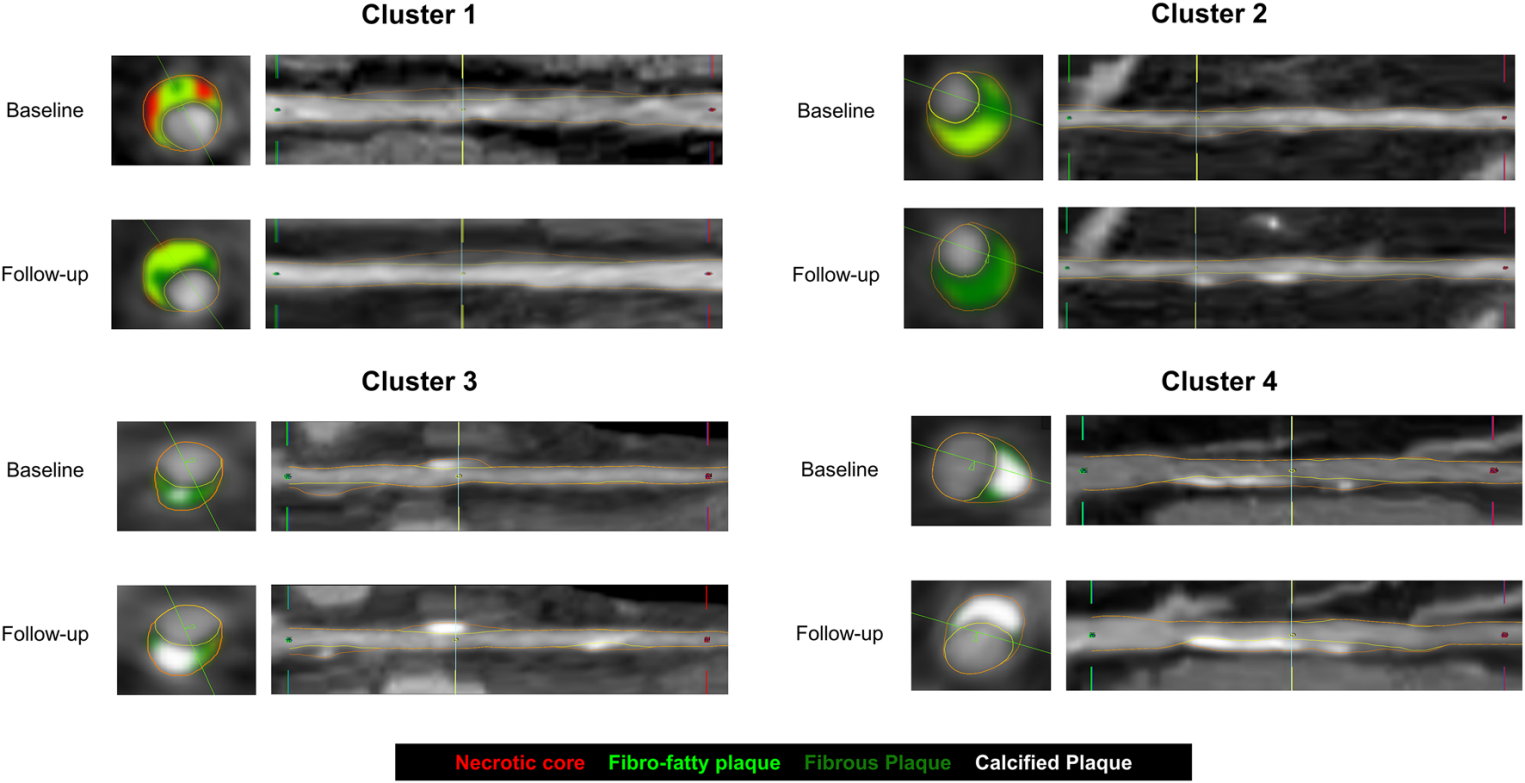
Representative cases from each cluster at baseline and follow-up.

*K*-means clustering was applied to 947 patients with coronary atherosclerotic plaque (age 62 years [56–67], 59% male) (Clinical characteristics in Supplementary Table 2) revealed 4 different groups of patients with significantly different plaque composition (*P* < 0.001 for all 4 plaque components) (Fig. 1). Cluster 1 (n = 52) mainly comprised fibro-fatty plaque (55.7% [47.2–61.8]), with a significant portion of necrotic core (16.0% [13.0–21.9]); Cluster 2 (n = 219) mainly comprised fibro-fatty plaque (39.2% [32.0–48.3]) and fibrous plaque (46.8% [40.0–53.5]); Cluster 3 (n = 376) mainly comprised fibrous plaque (62.7% [55.8–71.2]), with a significant portion of calcified plaque (23.6% [12.2–33.3]); and Cluster 4 (n = 300) mainly comprised calcified plaque (58.7% [49.9–71.3]). Representative cases of each cluster are shown in Fig. 2.

**Figure 3:**
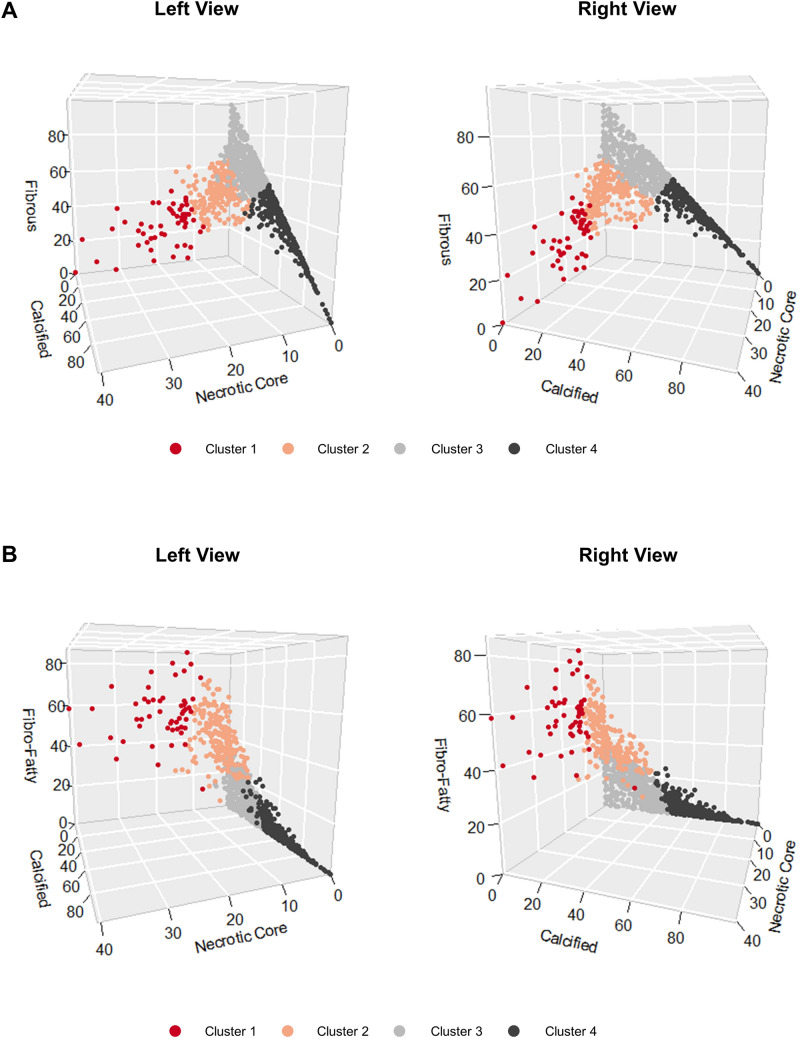
Three-dimensional (3D) plots visualizing the clusters. 3D plots visualizing the clusters (**A**) using the percent volumes of the necrotic core, fibrous plaque, and calcified plaque, and (**B**) using the percent volumes of the necrotic core, fibro-fatty plaque, and calcified plaque.

When we visualized clusters in 3D space (Fig. 3), the separation of Cluster 1 was mainly driven by its higher %vol of the necrotic core, and Cluster 4 was separated from others due to its higher calcified plaque %vol. Although Cluster 2 was separated from Clusters 3 and 4 by its higher %vol of fibro-fatty plaque, separation from Cluster 1 depended on its %vol of the necrotic core and fibrous plaque. We have also provided 2D plots using RadViz and t-SNE (Supplementary Fig. 3).

### Clinical characteristic comparisons

Clusters 1, 2, and 3 demonstrated quite similar clinical characteristics (Table 1). However, the patients in Cluster 4 tended to be older, have lower body mass index and triglyceride levels, and higher high-density lipoprotein levels than the patients in other clusters. While statin use at baseline was higher in Cluster 4 than in Cluster 3, statin use at follow-up was similar among clusters.

**Table 1:** Clinical characteristics.

	Cluster				*P*
	1	2	3	4	
	(N = 52)	(N = 219)	(N = 376)	(N = 300)	
Age, years	59 [54;65]^§^	60 [53;65]^§^	62 [55;67] ^§^	65 [59;70]*^†‡^	< 0.001
Men	35 (67%)	140 (64%)^§^	233 (62%)	155 (52%)^†^	0.009
10-year ASCVD risk, %	11.2 [5.3;20.3]	9.8 [5.2;17.2]^§^	10.3 [5.4;18.1]	12.1 [6.4;21.5]^†^	0.028
Body mass index, kg/m^2^	25.6 [24.2;27.4]^§^	25.0 [23.3;27.7]^§^	25.3 [23.7;27.5]^§^	24.3 [22.5;26.5]*^†‡^	< 0.001
Hypertension	32 (62%)	121 (55%)	209 (56%)	173 (58%)	0.790
Diabetes mellitus	18 (35%)	55 (25%)	83 (22%)	76 (25%)	0.242
Hyperlipidemia	10 (19%)^§^	71 (32%)^§^	144 (38%)	134 (45%)*^†^	0.001
Family history of CAD	12 (23%)	44 (20%)	103 (27%)	79 (26%)	0.228
Current smoking	15 (29%)	46 (21%)	72 (19%)	45 (15%)	0.077
Systolic blood pressure, mmHg	128 [120;140]	127 [120;140]	130 [119;140]	130 [119;140]	0.845
Diastolic blood pressure, mmHg	80 [70;84]	80 [70;84]	80 [72;85]^§^	76 [70;84]^‡^	0.033
**Lipid profile at baseline**					
Total cholesterol, mg/dl	188 [154;210]	184 [162;212]	190 [165;217]	186 [159;209]	0.168
Triglyceride, mg/dl	156 [114;184]^§^	138 [96;190]^§^	130 [92;186]^§^	117 [84;163]*^†‡^	0.001
HDL, mg/dl	45 [38;54]	46 [38;54]^§^	48 [41;57]^§^	51 [43;61]^†‡^	< 0.001
LDL, mg/dl	110 [91;134]	116 [93;142]	116 [90;139]	110 [87;132]	0.087
**Lipid profile at follow-up**					
Total cholesterol, mg/dl	159 [142;190]	166 [144;190]	168 [142;195]	164 [142;188]	0.612
Triglyceride, mg/dl	124 [88;186]^§^	116 [84;163]^§^	116 [82;164]^§^	104 [75;138]*^†‡^	0.004
HDL, mg/dl	48 [41;53]	45 [39;52]^‡§^	47 [40;57]^†^	51 [42;59]^†^	< 0.001
LDL, mg/dl	88 [70;110]	93 [72;120]	92 [76;118]	92 [74;120]	0.729
Statin at baseline	18 (35%)	94 (43%)	144 (38%)^§^	158 (53%)^‡^	0.001
Statin at follow-up	35 (67%)	140 (64%)	244 (65%)	221 (74%)	0.054

Data are presented as the median (interquartile range) for continuous variables and number (percentage) for categorical variables. Adjusted *P*-values for multiple comparisons < 0.05 are marked as * (vs. Cluster 1), † (vs. Cluster 2), ‡ (vs. Cluster 3), and § (vs. Cluster 4).

ASCVD = atherosclerotic cardiovascular disease, CAD = coronary artery disease, HDL = high-density lipoprotein, LDL = low-density lipoprotein.

### Baseline CCTA characteristic comparison

At baseline, Cluster 2 demonstrated the highest total PV (107.0 mm^3^ [42.3–194.1]) and Cluster 3 demonstrated the lowest total PV (63.9 mm^3^ [25.8–158.5]) (Table 2). Necrotic core and fibro-fatty PV were greatest in Cluster 1 and gradually decreased (in order) from Cluster 2 to Cluster 4 (*P* < 0.001 for both). Fibrous PV of Cluster 2 was comparable to that of Cluster 3 (*P* = 0.589) and Cluster 4 (P = 0.088) and was significantly greater than that of Cluster 1 (*P* = 0.001). The calcified PV was lowest in Cluster 1 and gradually increased (in order) from Cluster 2 to Cluster 4 (*P* < 0.001). Cluster 4 demonstrated the greatest maximal diameter and area stenosis (*P* < 0.001 for both).

**Table 2:** CCTA characteristics.

	Cluster				*P*
	1	2	3	4	
	(N = 52)	(N = 219)	(N = 376)	(N = 300)	
**Baseline CCTA characteristics**					
Total vessel length, mm	402 [351;440]	411 [ 336;489]	418 [342;487]	398 [309;485]	0.244
Total vessel volume, mm^3^	2121 [1747;2788]	2220 [1676;2867]	2247 [1683;3029]	2111 [1527;3023]	0.474
Plaque volume, mm^3^					
Total plaque	87.5 [55.5;141.5]	107.0 [42.3;194.1]^‡^	63.9 [25.8;158.5]^†§^	93.9 [39.8;257.2]^‡^	< 0.001
Necrotic core	17.0 [9.1;29.2]^†‡§^	3.6 [0.9; 8.3]*^‡§^	0.1 [0.0; 0.7]*^†§^	0.0 [0.0; 0.3]*^†‡^	< 0.001
Fibro-fatty plaque	45.4 [30.6;82.7]^‡§^	38.3 [17.0;74.5]^‡§^	7.6 [1.8;24.1]*^†§^	2.4 [0.2; 9.8]*^†‡^	< 0.001
Fibrous plaque	19.7 [10.4;41.9]^†‡§^	43.3 [20.9;85.3]*	38.0 [16.9;92.5]*	32.0 [13.1;84.7]*	< 0.001
Calcified plaque	0.0 [0.0;1.6]^†‡§^	3.9 [0.2;21.5]*^‡§^	13.4 [3.1;34.9]*^†§^	55.4 [21.6;152.9]*^†‡^	< 0.001
Maximal diameter stenosis, %	22.0 [14.4;32.1]^§^	24.3 [15.4;32.4]^§^	23.1 [13.5;32.4] ^§^	27.6 [18.1;37.9] *^†‡^	< 0.001
Maximal area stenosis, %	39.2 [26.7;53.9] ^§^	42.7 [28.4;54.3] ^§^	40.8 [25.1;54.3] ^§^	47.5 [32.9;61.4] *^†‡^	< 0.001
Number of vessels with obstructive disease					
None	48 (92.3%)^‡§^	202 (92.2%)*^‡§^	324 (86.2%)^†§^	246 (82.0%)*^†‡^	0.024
1	3 (5.8%)	12 (5.5%)	40 (10.6%)	32 (10.7%)	
2	0 (0.0%)	2 (0.9%)	8 (2.1%)	14 (4.7%)	
3	1 (1.9%)	3 (1.4%)	4 (1.1%)	8 (2.7%)	
Segment involvement score	2.0 [1.0;3.0]^†‡§^	3.0 [2.0;5.0]*^§^	3.0 [2.0;5.0]*^§^	4.0 [2.0;7.0] *^†‡^	< 0.001
Segment stenosis score	3.0 [2.0;4.0]^†‡§^	4.0 [2.0;7.0]*^§^	4.0 [2.0;7.0]*^§^	6.0 [3.0;10.0]*^†‡^	< 0.001
**Changes in CCTA characteristics at follow-up**					
CCTA interval, years	3.6 [2.6;5.2]	3.3 [2.6;4.8]	3.2 [2.6;4.6]	3.2 [2.6;4.5]	0.551
Change in plaque volume, mm^3^					
Total plaque	23.3 [− 7.5;79.6]^‡§^	37.9 [11.8;85.8]^§^	41.0 [11.5;105.6]*	50.7 [19.4;105.1]*^†^	< 0.001
Necrotic core	− 5.7 [− 17.6;− 0.3]^†‡§^	− 0.3 [− 3.6; 1.8]*^‡§^	0.0 [− 0.0; 0.9]*^†§^	0.0 [− 0.0; 0.2]*^†‡^	< 0.001
Fibro-fatty plaque	− 5.6 [− 28.2;14.9]^‡§^	− 2.4 [− 22.4;12.7]^‡§^	0.1 [− 4.2; 7.9]*†	0.0 [− 1.9; 4.3]*^†^	< 0.001
Fibrous plaque	26.5 [15.3;55.0]^‡§^	18.5 [6.3;48.3]^‡§^	9.8 [− 1.6;36.0]*^†§^	5.4 [− 7.9;25.2]*^†‡^	< 0.001
Calcified plaque	2.5 [0.2;16.9]^†‡§^	13.0 [2.4;35.1]*^‡§^	21.4 [5.9;52.7]*^†§^	35.9 [14.4;88.7]*^†‡^	< 0.001
Annualized change in plaque, mm^3^/year					
Total plaque	5.7 [− 1.6;22.0]^‡§^	10.1 [3.1;21.5]^§^	11.6 [3.4;32.9]*^†^	14.4 [5.8;31.6]*^†^	< 0.001
**Other parameters at follow-up**					
Maximal diameter stenosis, %	25.6 [18.8;35.2]^§^	28.7 [20.1;37.6]^§^	26.6 [17.5;36.4]^§^	32.1 [23.0;41.7]*^†‡^	< 0.001
Maximal area stenosis, %	44.7 [34.0;58.0]^§^	49.1 [36.1;61.1]^§^	46.1 [32.0;59.5]^§^	53.9 [40.7;66.1]*^†‡^	< 0.001
No. of vessels with obstructive disease					
None	43 (82.7%)	180 (82.2%)	296 (78.7%)	215 (71.7%)	0.105
1	6 (11.5%)	29 (13.2%)	49 (13.0%)	52 (17.3%)	
2	1 (1.9%)	3 (1.4%)	16 (4.3%)	20 (6.7%)	
3	2 (3.8%)	7 (3.2%)	15 (4.0%)	13 (4.3%)	
Segment involvement score	3.0 [2.0;4.0]^†‡§^	4.0 [3.0;6.0]*^§^	4.0 [3.0;7.0]*^§^	5.0 [3.0;8.0]*^†‡^	< 0.001
Segment stenosis score	4.0 [2.0;5.0]^†‡§^	6.0 [3.0;9.0]*^§^	6.0 [3.0;10.0]*^§^	8.0 [5.0;12.0]*^†‡^	< 0.001

Data are presented as the median (interquartile range) for continuous variables and number (percentage) for categorical variables Adjusted *P*-values for multiple comparisons < 0.05 are marked as * (vs. Cluster 1), † (vs. Cluster 2), ‡ (vs. Cluster 3), and § (vs. Cluster 4).

CCTA = coronary computed tomographic angiography.

### Changes in CCTA characteristics at follow-up

At follow-up (3.3 years [2.6–4.7]), the change in total PV gradually increased (in order) from Cluster 1 to Cluster 4 (23.3 mm^3^ [-7.5–79.6], 37.9 mm^3^ [11.8–85.8], 41.0 mm^3^ [11.5–105.6], and 50.7 mm^3^ [19.4–105.1], respectively, *P* < 0.001) (Table 2). Necrotic core and fibro-fatty PV regression were evident in Cluster 1 and gradually weakened (in order) from Cluster 2 to Cluster 3 and Cluster 4 (*P* < 0.001 for both). While the increase in fibrous PV was highest in Cluster 1 and gradually decreased in order from Cluster 2, 3, and 4 (*P* < 0.001), the change in calcified PV showed a gradual increase from Cluster 1 to Cluster 4 (*P* < 0.001). On multivariable logistic regression analysis, the risk of plaque progression for Cluster 2 was comparable to that of Cluster 1 (aOR 1.56, 95% CI 0.79–3.17, *P* = 0.207), but was significantly higher for Clusters 3 and 4 than for Cluster 1 (aOR 2.53, 95% CI 1.32–5.02, *P* = 0.006; and aOR 2.43, 95% CI 1.25–4.87, *P* = 0.010, respectively) (Supplementary Table 3).

**Figure 4:**
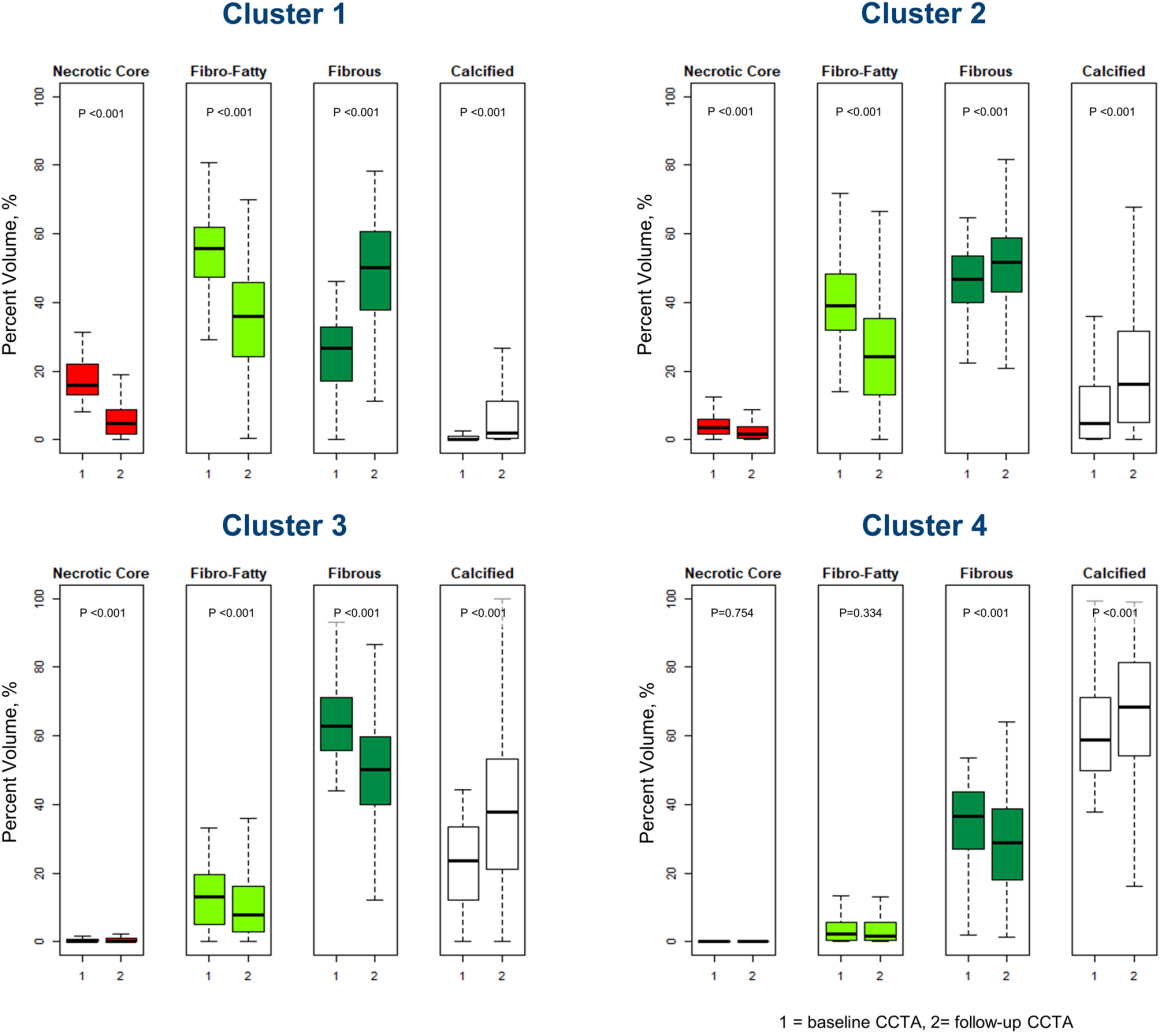
Each plaque component %vol at the time of baseline and follow-up CCTAs. Cluster 1 demonstrated decreased %vols of the necrotic core and fibro-fatty plaque, and increased %vols of fibrous plaque and calcified plaque. Although Cluster 2 showed a greater increase in the calcified portion than did Cluster 1, both clusters showed PV progression mainly driven by an increase in fibrous PV. In contrast, in Clusters 3 and 4, the fibrous plaque %vol decreased, and PV progression was mostly driven by an increase in calcified PV.

Each plaque component %vol at the time of baseline and follow-up CCTAs are illustrated in Fig. 4. Cluster 1, which had the least total PV progression, demonstrated decreased %vols of the necrotic core (median, from 16.0% to 4.8%, *P* < 0.001) and fibro-fatty plaque (from 55.7% to 35.9%, *P* < 0.001), and increased %vols of fibrous plaque (from 26.7% to 50.3%, *P* < 0.001) and calcified plaque (from 0.0% to 2.1%, *P* < 0.001). Although Cluster 2 showed a greater increase in the calcified portion (from 4.7% to 16.3%, *P* < 0.001) than did Cluster 1, both clusters showed PV progression mainly driven by an increase in fibrous PV (114% and 48% of the total PV increase, respectively). In contrast, in Clusters 3 and 4, the fibrous plaque %vol decreased (from 62.7% to 50.3%, *P* < 0.001; and from 36.4% to 28.9%, respectively, *P* < 0.001), and PV progression was mostly driven by an increase in calcified PV (52% and 71% of the total PV increase, respectively).

### Changes in plaque volume according to statin use

When we evaluated the differences in PV progression among clusters in statin-naïve patients (n = 307), there was no significant difference in the total PV change (*P* = 0.241) and necrotic core and fibro-fatty PV regression was not evident even in Cluster 1 (Supplementary Table 4). Whereas, in statin-taking patients (n = 640), necrotic core or fibro-fatty PV regression was observed in Cluster 1 and 2, and the total PV increase was significantly greater in Cluster 3 (50.2 mm^3^ [14.0–124.2]) and Cluster 4 (55.8 mm^3^ [21.5–130.8]) than in Cluster 1 (15.5 mm^3^ [-11.5–46.5]) and Cluster 2 (35.2 mm^3^ [9.8–94.3], *P* < 0.001). Further stratification results according to the low-density lipoprotein (LDL) level at follow-up showed the differences in PV progression across clusters were more evident in patients with well-controlled LDL levels (< 100 mg/dL) (Supplementary Table 5). Multivariable logistic regression analysis showed a higher risk of plaque progression for Clusters 3 and 4 in statin-taking patients, but not in statin-naïve patients (Supplementary Table 3).

### Clinical outcome comparisons

The incidence of MACE was significantly lower in Cluster 1 (6.1%) than in Cluster 2 (23.0%, *P* = 0.033), Cluster 3 (19.9%, *P* = 0.050), and Cluster 4 (22.6%, *P* = 0.033) (Supplementary Fig. 4). Multivariable Cox regression analysis showed a higher risk of MACE for Cluster 2 (aHR 4.48, 95% CI 1.39–14.45, *P* = 0.011), Cluster 3 (aHR 3.55, 95% CI 1.11–11.34, *P* = 0.032), and Cluster 4 (aHR 3.28, 95% CI 1.02–10.56, *P* = 0.046) than for Cluster 1 (Supplementary Table 6). Subgroup analysis according to statin use demonstrated an increased risk for MACE in Clusters 2, 3, and 4 than in Cluster 1 in statin-taking patients, but not in statin-naïve patients.

## Discussion

The present analysis of a large prospective observational cohort of patients with coronary atherosclerosis undergoing serial CCTA used unsupervised cluster analysis to categorize patients according to their coronary atherosclerotic plaque composition. The identified clusters of patients demonstrated markedly different plaque progression patterns, changes in composition, and clinical outcomes. This study provides insight into how patients with heterogeneous coronary atherosclerotic plaque composition differentially experience coronary atherosclerotic plaque progression and adverse cardiac events according to their baseline plaque composition.

CCTA enables the accurate assessment of the change in coronary atherosclerotic plaque noninvasively over time^1,19,20^. Recent advances have further promoted the use of CCTA, by providing semi-automated segmentation and characterization of the plaque composition. The PARADIGM registry is the largest available serial CCTA database with quantitative measures of atherosclerotic burden and composition^8^. The prior PARADIGM registry studies provided important information regarding the natural course of coronary atherosclerosis and the clinical determinants of plaque progression or regression, by evaluating the impact of statin taking or high-risk features on the progression of coronary plaque lesions^2,21^ or categorizing patients according to their clinical risk factor s such as diabetes mellitus^3,22^. In the present study, we performed patient-specific plaque phenotyping, using the ability of CCTA to visualize plaque components in the entire coronary tree, to bridge the gap between the recognition of heterogeneous plaque composition on CCTA and individualized cardiovascular risk assessment and preventive strategy establishment.

Unsupervised clustering is an exploratory data analysis technique that provides insight into the data structure by segregating groups with similar traits and assigning them into clusters^7,14^. *K-*means clustering is one of the most popular and simplest clustering algorithms. It partitions a feature space into *k* clusters by placing each data point in the cluster closest to its mean value^23^. Since we aimed to categorize heterogeneous patients with coronary atherosclerosis according to their baseline atherosclerotic plaque composition, we applied *k-*means clustering to the %vol of each plaque component. In other words, *k-*means clustering allowed us to find groups of similar data points in a 4-dimensional feature space comprising the %vols of the necrotic core, fibro-fatty plaque, fibrous plaque, and calcified plaque. Considering that coronary atherosclerosis is a continuous process, it was not surprising that the distances between clusters were not large. Nevertheless, the resulting 4 clusters demonstrated distinct features and significantly different plaque progression patterns.

Phenotyping coronary atherosclerotic plaque at the patient level offers insight into how the progression and transformation of coronary atherosclerosis differ according to the baseline composition. Hwang et al. previously applied topological data analysis (TDA) to PARADIGM registry data and identified three distinct group of patients^24^. Since TDA aims to pattern or shape the complex dataset using a geometric approach, multiple quantitative CCTA parameters, including total vessel length, total vessel volume, total lumen volume, PV, fibrous component volume, fibrofatty component volume, necrotic core volume, and dense calcium volume, were utilized to categorize patients in this study. The resultant groups demonstrated not only distinct plaque composition but also increasing PV accompanied by increasing age and prevalence of comorbidities. In contrast, the present clustering was performed independently of patient clinical characteristics and other CCTA characteristics, such as the total PV. Nevertheless, the resultant clusters from both studies are in concordance with the known natural history of atherosclerotic plaque^24^, suggesting that the clustering was in accordance with the evolutionary stage of atherosclerosis at the patient level^25–27^. The present study provides deeper insight into the compositional changes during the plaque progression. Clusters 1 and 2 comprised patients who had earlier-stage coronary atherosclerotic plaques with more vulnerable plaque components, such as the necrotic core and fibro-fatty plaque, showed regression of these components and PV progression mainly driven by an increase in fibrous PV. Clusters 3 and 4 represented patients who had more advanced and stabilized plaques, with more calcium and showed PV progression mostly driven by an increase in calcified PV. The similarities in clinical characteristics between clusters can be attributed to the multifactorial influences on the advent and progression of coronary atherosclerosis in an individual. Nevertheless, Clusters 3 and 4 demonstrated a higher risk for plaque progression, independent of clinical risk factors, statin use, and baseline total PV. The differential progression status among clusters underlines the role of CCTA in evaluating plaque composition, in addition to obstruction severity and plaque burden. Furthermore, the significantly different risk for MACE between clusters suggests that patient-specific phenotyping of coronary atherosclerosis would facilitate personalized risk assessment and preventive treatment.

The ability to predict how coronary atherosclerotic plaque progresses based on its composition may help clinicians decide who may benefit most from statins or other modifiers of atherosclerotic pathogenesis, while reducing harm. We additionally evaluated whether statin use differentially affects the plaque progression according to the baseline plaque composition. Although the PARADIGM registry’s observational study design limits the direct comparison of the impact of statin use in each cluster, the subgroup analysis according to statin use provided clues regarding the differential impact of statins across clusters. The higher risk of plaque progression in Clusters 3 and 4 compared to Cluster 1 was only observed in statin-taking patients, not in statin-naïve patients. Similarly, the higher risk of MACE in Clusters 2, 3, and 4 was only observed in statin-taking patients. The observation of preserved differential plaque progression and clinical outcome in only statin-taking patients supports the need for a more personalized assessment of the cardiovascular risk and the deployment of a preventive strategy based on patient-specific plaque phenotyping. However, the value of patient-specific plaque phenotyping in facilitating personalized decision-making regarding statin use should be evaluated in randomized controlled trials that integrate CCTA with a targeted prevention strategy.

### Study limitations

First, the PARADIGM study enrolled patients with repeated CCTA scans. Therefore, the current study populations mostly comprised patients with low-to-moderate risk, and were, therefore, not eligible for invasive coronary angiography. Furthermore, as patients with more rapid progression were more likely to experience clinical events and might not attend a second CCTA, the study population tended to represent patients with earlier stage of coronary artery disease; the risk of this selection bias must be considered before generalizing these results to a higher-risk population. However, our results provide valuable clues regarding earlier changes in coronary atherosclerosis. The difference in plaque progression and MACE risk across the clusters indicates that the evaluation of plaque composition using CCTA has clinical implication from the earlier stage of coronary atherosclerosis. Second, the optimal number of clusters in *k*-means clustering is somewhat subjective; however, our decision to use 4 clusters was based on the Calinski-Harabasz Index and Average Silhouette Width, as well as the elbow method^28^. Furthermore, the visualization of the clusters suggests that the clustering was done in a clinically intuitive manner based on the %vols of the 4 different plaque components. Finally, although an external validation dataset was not available, because of the paucity of registries similar to the PARADIGM registry with serial and quantitative measures of each plaque component, the clustering algorithm provided stable phenotyping as supported by bootstrapping validation. Ideal clustering should not only have good statistical properties, but should also provide clinically relevant results. We believe that the current study results provide important clues to understanding the impact of patient-level plaque composition on plaque progression and change in its character.

## Conclusion

In conclusion, unsupervised clustering analysis of patients with coronary atherosclerotic plaque identified substantial phenotypic heterogeneity in coronary atherosclerotic plaque composition. Patient-specific plaque phenotyping may help our understanding of heterogeneity in coronary atherosclerotic plaque progression. Further research is needed to determine the utility of patient-specific plaque phenotyping in personalized risk assessment and preventive treatment.

## Supplementary Information

Supplementary Information.
