# Supplementary material for: Differential progression of coronary atherosclerosis according to plaque composition: a cluster analysis of PARADIGM registry data
Source: Sci Rep. 2021 Aug 24;11:17121. doi: 10.1038/s41598-021-96616-w (PMC8385056; doi:10.1038/s41598-021-96616-w)
Supplement: Supplementary file 1 — Supplementary Information. [file 41598_2021_96616_MOESM1_ESM.doc]

**Differential Progression of Coronary Atherosclerosis According to Plaque Composition**

**A Cluster Analysis of PARADIGM Registry Data**

**Supplementary Data**

**Page**

**Supplementary Figure 1. Study flow**  2

**Supplementary Figure 2. Determination of the number of clusters** 3

**Supplementary Figure 3. Visualization of the clusters in 2-Dimensional (2D) plots 4**

**Supplementary Figure 4. The occurrence of major adverse cardiac events (MACE) according to cluster** 5

**Supplementary Table 1. Cluster stability measures** 6

**Supplementary Table 2. Baseline characteristics of entire study population** 7

**Supplementary Table 3. Multivariable logistic regression analysis**  8

**Supplementary Table 4. Changes in plaque volume (PV) across clusters according to statin taking**  9

**Supplementary Table 5. Changes in plaque volume (PV) across clusters according to the LDL level at follow-up in statin-taking patients** 10

**Supplementary Table 6. Multivariable Cox regression analysis** 11

**Supplementary Figure 1. Study flow**


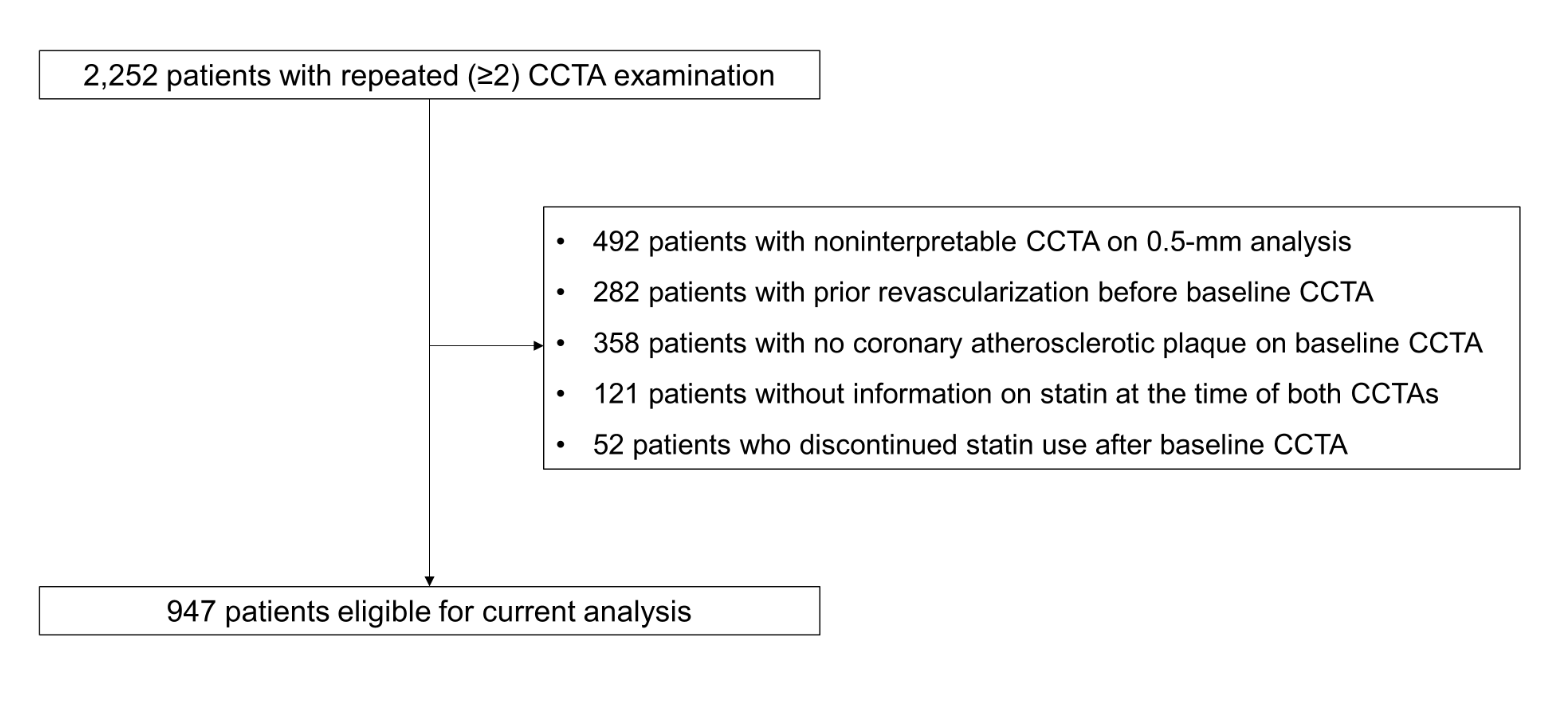


CCTA = coronary computed tomographic angiography

**Supplementary Figure 2. Determination of the number of clusters. (A) Plots of the total within-cluster sum of squares (WCSS), and (B) the Calinski-Harabasz index (ch) and average silhouette width (asw), for the number of clusters (k) in the range of 1 to 10.**

The elbow method looks at the total WCSS as a function of the number of clusters (k). The location of a bend in the plot is a heuristic suggesting diminishing returns. It is considered an indicator of the optimal number of clusters, and is the threshold for identifying most of the variation. However, the elbow method is sometimes ambiguous, as it is in this plot (k from 3 to 6 could be selected). Therefore, we additionally estimated the ch, which is the ratio of the between-cluster variance to the total WCSS, and asw, which measures how well an individual data point is clustered, and further estimated the average distance between clusters. While ch was highest when k was 3, the asw was highest when k was 6. Finally, we decided on 4 as the optimal number of clusters, in consideration of all these parameters.


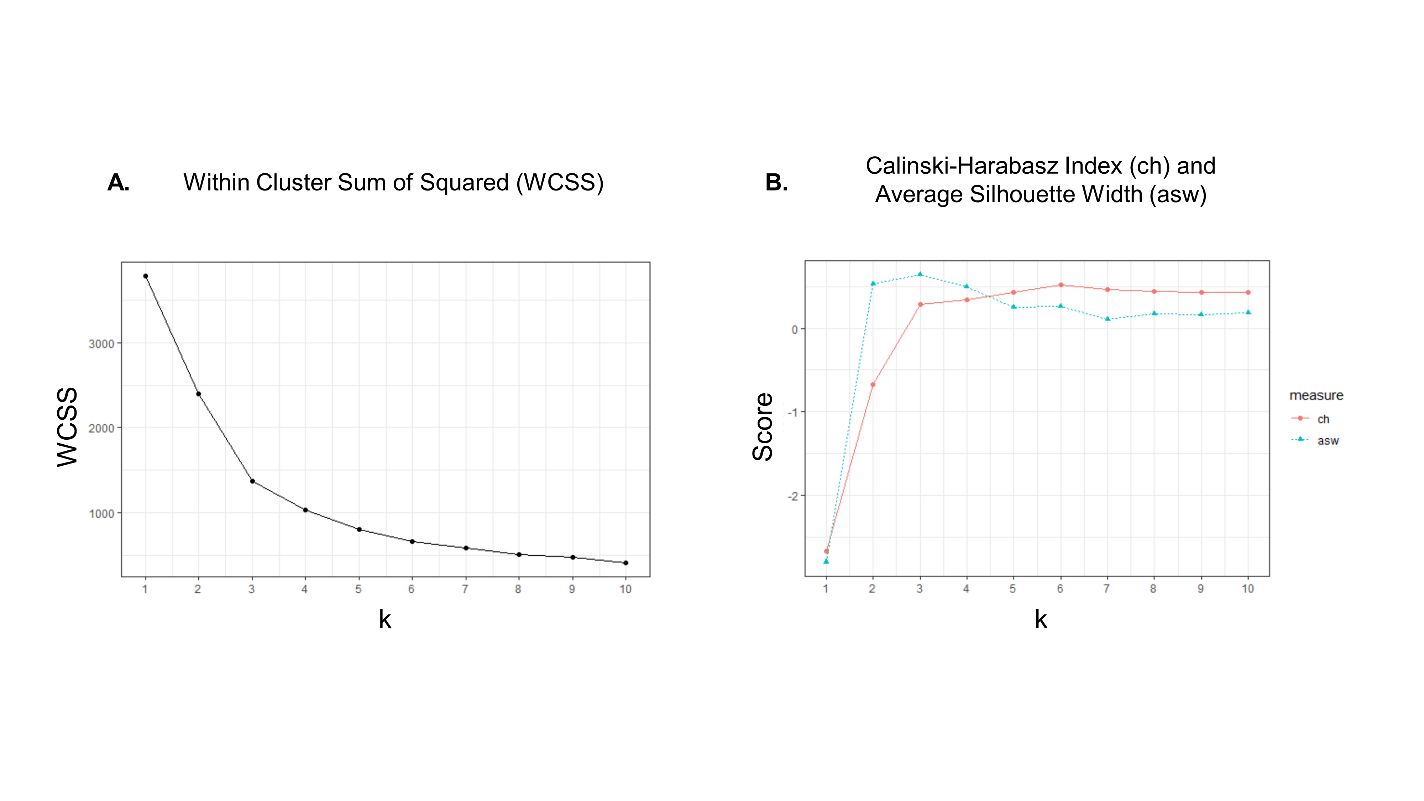


**Supplementary Figure 3. Visualization of the clusters in 2-Dimensional (2D) plots using (A) radial visualization (RadViz), and (B) t-distributed stochastic neighbour embedding (t-SNE).**

RadViz and t-SNE provide visualization of data defined by 3 or more variables in a 2D projection. In RadViz, each dimension in the dataset is represented by a dimensional anchor, and each dimensional anchor is distributed evenly on a unit circle. RadiViz plots normalized data points along each axis to lie within the circle. In contrast, t-SNE calculates the probability of the similarity of data points in a higher-dimensional space and the probability of the similarity of points in the corresponding lower-dimensional space; t-SNE then minimizes the differences between similarities in both spaces to ultimately represent the data points in a lower-dimensional space.


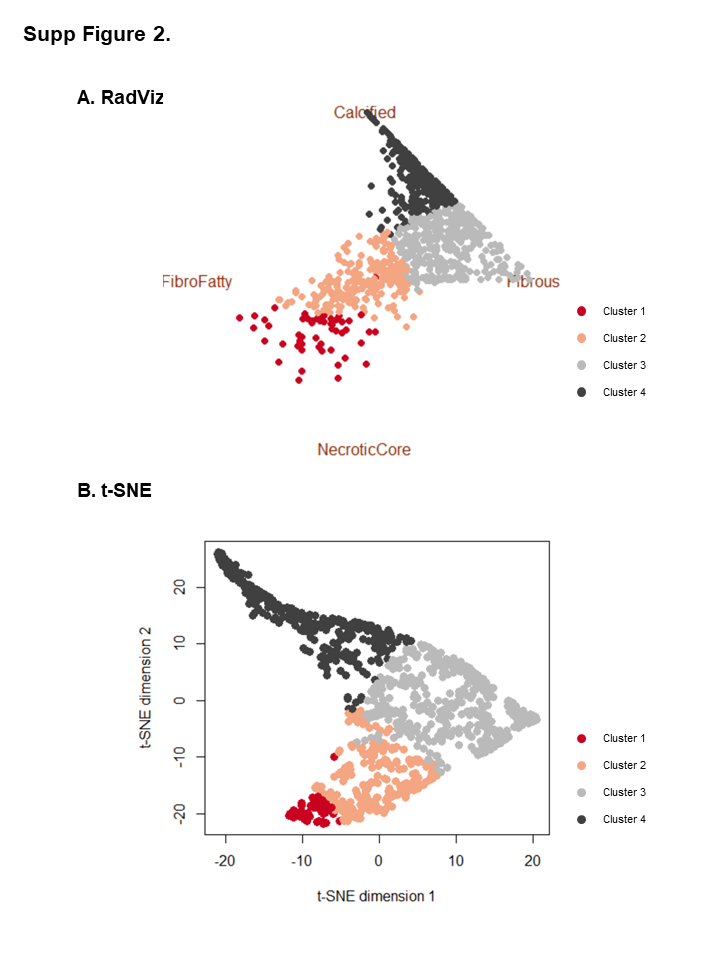


**Supplementary Figure 4. The occurrence of major adverse cardiac events (MACE) according to cluster.**

MACE was defined as the composite of all-cause death, acute coronary syndrome, and coronary revascularization. MACE-free survival data were plotted using the Kaplan-Meier method and compared by using the log-rank test.

**A. Incidence of MACE after baseline coronary computed tomographic angiography according to cluster**

|  | **Cluster** | | | |
| --- | --- | --- | --- | --- |
|  | **1** | **2** | **3** | **4** |
|  | **(N=49)** | **(N=196)** | **(N=311)** | **(N=252)** |
| MACE | 3 (6.1%) | 45 (23.0%) | 62 (19.9%) | 57 (22.6%) |
|  |  |  |  |  |
| All cause death | 0 (0.0%) | 1 (0.5%) | 4 (1.3%) | 6 (2.4%) |
| Acute coronary syndrome | 0 (0.0%) | 1 (0.5%) | 4 (1.3%) | 4 (1.6%) |
| Coronary revascularization | 3 (6.1%) | 43 (21.9%) | 54 (17.4%) | 47 (18.7%) |

**B. Kaplan-Meier MACE-free survival curves**


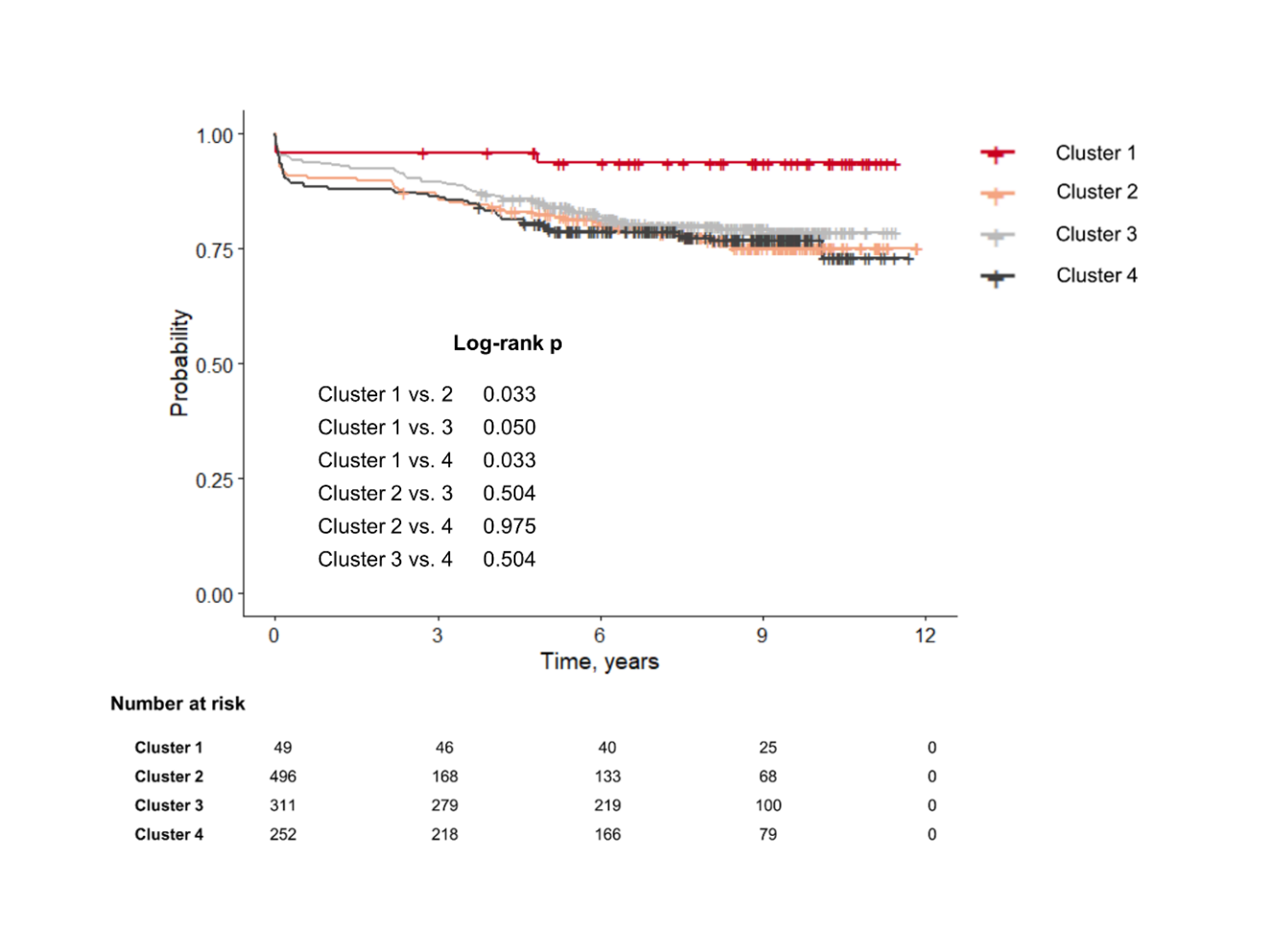


**Supplementary Table 1. Cluster stability measures.**

Cluster stability was validated using nonparametric bootstrapping (100 resamples with 100 iterations). The clusters formed by bootstrapping were the same as the initially formed clusters. We also computed the cluster-wise Jaccard coefficient and the number of dissolutions. All values from the analysis support the stability of the current clustering.

| Cluster | 1 | 2 | 3 | 4 |
| --- | --- | --- | --- | --- |
| Jaccard coefficient | 0.84 | 0.89 | 0.95 | 0.99 |
| Number of dissolutions | 0.00 | 0.00 | 0.00 | 0.00 |

**Supplementary** Table 2. Baseline characteristics of entire study population

|  | **Entire Population**  (N=947) |
| --- | --- |
| Age, years | 62 [56;67] |
| Men | 563 (59%) |
| 10-year ASCVD risk, % | 10.7 [5.6;19.6] |
| BMI, kg/m^2^ | 25.0 [23.3;27.2] |
| Hypertension | 535 (56%) |
| Diabetes mellitus | 232 (25%) |
| Hyperlipidemia | 359 (38%) |
| Family history of CAD | 238 (25%) |
| Current smoking | 178 (19%) |
| SBP, mmHg | 130 [120;140] |
| DBP, mmHg | 80 [70;85] |
| Lipid profile at baseline |  |
| Total cholesterol, mg/dl | 187 [162;213] |
| Triglyceride, mg/dl | 128 [91;183] |
| HDL, mg/dl | 48 [40;58] |
| LDL, mg/dl | 114 [90;138] |
| Lipid profile at follow-up |  |
| Total cholesterol, mg/dl | 166 [142;191] |
| Triglyceride, mg/dl | 111 [79;157] |
| HDL, mg/dl | 47 [41;57] |
| LDL, mg/dl | 92 [74;119] |
| Statin at baseline | 414 (44%) |
| Statin at follow-up | 640 (68%) |

ASCVD=atherosclerotic cardiovascular disease, BMI=body mass index, CAD=coronary artery disease, DBP=diastolic blood pressure, HDL=high-density lipoprotein, LDL=low-density lipoprotein, SBP=systolic blood pressure

**Supplementary Table 3. Multivariable logistic regression analysis.**

Multivariable logistic regression analysis was performed to compare the risk of plaque progression among clusters. The 10-year atherosclerotic cardiovascular disease (ASCVD) risk, diabetes mellitus, total plaque volume (PV) at baseline, and statin use were included in the multivariable analysis. The results are expressed as the adjusted odds ratio (aOR) and the corresponding 95% confidence interval (CI).

|  | Entire population (n=947) | | | | |  | Statin-naïve patients (n=307) | | | | |  | Statin-taking patients (n=640) | | | | |
| --- | --- | --- | --- | --- | --- | --- | --- | --- | --- | --- | --- | --- | --- | --- | --- | --- | --- |
|  | aOR | 95% CI | | | *P* |  | aOR | 95% CI | | | *P* |  | aOR | 95% CI | | | *P* |
| 10-year ASCVD risk | 1.00 | 0.99 | - | 1.02 | 0.674 |  | 1.03 | 1.00 | - | 1.06 | 0.047 |  | 0.99 | 0.97 | - | 1.01 | 0.352 |
| Diabetes mellitus | 1.93 | 1.35 | - | 2.78 | <0.001 |  | 1.23 | 0.57 | - | 2.67 | 0.594 |  | 2.18 | 1.44 | - | 3.32 | <0.001 |
| Total PV at baseline | 1.01 | 1.01 | - | 1.01 | <0.001 |  | 1.01 | 1.01 | - | 1.02 | <0.001 |  | 1.01 | 1.01 | - | 1.01 | <0.001 |
| Statin use | 0.81 | 0.60 | - | 1.10 | 0.178 |  | - | - |  | - | - |  | - | - |  | - | - |
| Cluster 1 (reference) | 1.00 | - |  | - | - |  | 1.00 | - |  | - | - |  | 1.00 | - |  | - | - |
| Cluster 2 | 1.56 | 0.79 | - | 3.17 | 0.207 |  | 1.45 | 0.43 | - | 5.13 | 0.552 |  | 1.55 | 0.66 | - | 3.21 | 0.325 |
| Cluster 3 | 2.53 | 1.32 | - | 5.02 | 0.006 |  | 1.64 | 0.52 | - | 5.59 | 0.412 |  | 3.35 | 1.50 | - | 8.04 | 0.004 |
| Cluster 4 | 2.43 | 1.25 | - | 4.87 | 0.010 |  | 2.20 | 0.67 | - | 7.72 | 0.200 |  | 2.68 | 1.18 | - | 6.46 | 0.022 |

**Supplementary Table 4. Changes in plaque** volume (PV) across clusters according to statin taking

|  | **Cluster in Statin-Naïve Patients (N=307)** | | | | ***P*** |
| --- | --- | --- | --- | --- | --- |
|  | **1** | **2** | **3** | **4** |  |
|  | **(N=17)** | **(N=79)** | **(N=132)** | **(N=79)** |  |
| CCTA interval, years | 2.9 [2.4; 4.8] | 3.1 [2.5;4.5] | 3.1 [2.5;3.9] | 3.1 [2.4;4.4] | 0.904 |
| Change in PV, mm^3^ |  |  |  |  |  |
| Total plaque | 27.1 [2.6;84.9] | 40.4 [15.7;73.9] | 27.0 [7.7;68.3] | 38.2 [17.6;75.7] | 0.241 |
| Necrotic core | -1.2 [-5.3; 5.8] | 0.0 [-2.3; 1.8] | 0.0 [0.0; 1.0] | 0.0 [ 0.0; 0.2] | 0.130 |
| Fibro-fatty plaque | 11.4 [-13.5;71.5] | -0.3 [-10.9;14.2] | 0.2 [-1.4; 7.4] | 0.5 [-0.2; 4.7] | 0.560 |
| Fibrous plaque | 25.5 [14.4;38.4]^‡§^ | 19.0 [8.0;41.3]^‡§^ | 9.8 [-0.9;29.3]^*†^ | 10.0 [-3.5;25.2]^*†^ | 0.001 |
| Calcified plaque | 0.6 [0.0; 4.3]^†‡§^ | 12.1 [2.1;28.3]^*§^ | 12.1 [4.3;33.3]^*§^ | 23.8 [8.7;58.4]^*†‡^ | <0.000 |
|  | **Cluster in Statin-Taking Patients (N=640)** | | | | ***P*** |
|  | **1** | **2** | **3** | **4** |  |
|  | **(N=35)** | **(N=140)** | **(N=244)** | **(N=221)** |  |
| CCTA interval, years | 4.1 [2.8;5.8] | 3.4 [2.6;4.9] | 3.3 [2.6;4.8] | 3.3 [2.6;4.6] | 0.381 |
| Change in PV, mm^3^ |  |  |  |  |  |
| Total plaque | 15.5 [-11.5;46.5]^‡§^ | 35.2 [9.8;94.3]^‡§^ | 50.2 [14.0;124.2]^*†^ | 55.8 [21.5;130.8]^*†^ | <0.001 |
| Necrotic core | -9.2 [-19.4;-3.4]^†‡§^ | -0.6 [-4.3; 1.8]^*‡§^ | 0.0 [-0.1; 0.8]^*†^ | 0.0 [-0.1; 0.2]^*†^ | <0.001 |
| Fibro-fatty plaque | -8.8 [-33.6; 3.0]^‡§^ | -5.4 [-29.5; 9.8]^‡§^ | 0.0 [-6.6; 8.3]^*†^ | 0.0 [-2.4; 4.2]^*†^ | <0.001 |
| Fibrous plaque | 27.5 [17.1;57.6]^‡§^ | 17.5 [5.9;53.7]^‡§^ | 9.8 [-2.5;38.2]^*†§^ | 3.7 [-11.0;23.8]^*†‡^ | <0.001 |
| Calcified plaque | 9.1 [0.8;20.7]^†‡§^ | 15.3 [2.8;38.1]^*‡§^ | 28.9 [ 8.0;61.8]^*†§^ | 44.1 [17.7;100.8]^*†‡^ | <0.001 |

Data are presented as the median (interquartile range) for continuous variables and number (percentage) for categorical variables. Adjusted *P*-values for multiple comparisons <0.05 are marked as * (vs. Cluster 1), † (vs. Cluster 2), ‡ (vs. Cluster 3), and § (vs. Cluster 4).

CCTA = coronary computed tomographic angiography, PV = plaque volume

**Supplementary Table 5. Changes in plaque volume (PV) across clusters according to the LDL level at follow-up in statin-taking patients**

Further stratification was performed in statin-taking patients with available LDL data at the time of follow-up (580 of 640 patients, 90.6%).

|  | LDL at follow-up ≥100mg/dL | | | |  |
| --- | --- | --- | --- | --- | --- |
|  | 1 | 2 | 3 | 4 | p |
|  | (N=7) | (N=42) | (N=65) | (N=66) |  |
| CCTA interval, years | 4.4 [ 3.3; 6.3] | 3.9 [ 3.0; 5.3] | 3.2 [ 2.4; 5.0] | 3.3 [ 2.6; 4.7] | 0.200 |
| Change in PV, mm^3^ |  |  |  |  |  |
| Total plaque | 15.9 [-5.2;80.8] | 37.6 [ 9.6;70.5] | 40.0 [13.2;89.8] | 54.1 [23.7;98.6] | 0.231 |
| Necrotic core | -9.2 [-18.3; 0.2] | -0.6 [-5.2; 0.5]^‡^ | 0.0 [-0.0; 0.4]^†^ | 0.0 [-0.1; 0.1] | 0.006 |
| Fibro-fatty plaque | -9.3 [-26.5;-7.3]^‡^ | -8.5 [-29.7; 6.4]^‡§^ | 0.2 [-1.8; 7.4]^*†^ | 0.0 [-3.5; 3.4]^†^ | 0.001 |
| Fibrous plaque | 17.9 [10.5;83.5] | 17.9 [ 6.0;51.1]^§^ | 9.5 [-1.0;34.2] | 7.0 [-11.9;27.1]^†^ | 0.018 |
| Calcified plaque | 20.4 [10.1;24.9] | 22.2 [ 3.3;37.4]^§^ | 21.8 [ 8.5;47.7]^§^ | 40.0 [17.7;80.0]^†‡^ | 0.002 |
|  | LDL at follow-up <100mg/dL | | | |  |
|  | 1 | 2 | 3 | 4 | p |
|  | (N=27) | (N=90) | (N=152) | (N=131) |  |
| CCTA interval, years | 4.1 [ 2.7; 5.5] | 3.3 [ 2.5; 4.8] | 3.3 [ 2.7; 4.9] | 3.4 [ 2.6; 4.6] | 0.503 |
| Change in PV, mm^3^ |  |  |  |  |  |
| Total plaque | 15.5 [-11.5;46.5]^‡§^ | 33.3 [ 8.6;99.9]^§^ | 58.7 [13.7;143.7]^*^ | 62.2 [21.5;144.6]^*†^ | 0.001 |
| Necrotic core | -10.1 [-19.4;-4.1]^†‡§^ | -0.7 [-3.1; 1.8]^*‡§^ | 0.0 [-0.3; 1.0]^*†^ | 0.0 [-0.1; 0.2]^*†^ | <0.001 |
| Fibro-fatty plaque | -7.0 [-33.6; 3.0]^‡§^ | -5.0 [-29.4;14.9]^‡§^ | -0.2 [-7.9;10.0]^*†^ | 0.0 [-2.2; 4.4]^*†^ | 0.002 |
| Fibrous plaque | 28.1 [18.0;57.6]^‡§^ | 17.7 [ 5.8;57.0]^‡§^ | 9.5 [-2.8;41.5]^*†^ | 4.6 [-9.9;29.1]^*†^ | <0.001 |
| Calcified plaque | 2.9 [ 0.6;16.0]^†‡§^ | 12.1 [ 2.2;39.0]^*‡§^ | 33.3 [ 7.4;62.8]^*†§^ | 49.2 [18.7;110.8]^*†‡^ | <0.001 |

Data are presented as the median (interquartile range) for continuous variables and number (percentage) for categorical variables. Adjusted *P*-values for multiple comparisons <0.05 are marked as * (vs. Cluster 1), † (vs. Cluster 2), ‡ (vs. Cluster 3), and § (vs. Cluster 4).

CCTA = coronary computed tomographic angiography, PV = plaque volume; LDL = Low-density lipoprotein

**Supplementary Table 6. Multivariable Cox regression analysis.**

Multivariable Cox regression analysis was performed to compare the risk of major adverse cardiac events (MACE) among clusters. The 10-year atherosclerotic cardiovascular disease (ASCVD) risk, diabetes mellitus, total plaque volume (PV) at baseline, annualized change in total PV, and statin use were included in the multivariable analysis. The results are expressed as the adjusted hazard ratio (aHR) and the corresponding 95% confidence interval (CI).

|  | Entire population (n=947) | | | | |  | Statin-naïve patients (n=307) | | | | |  | Statin-taking patients (n=640) | | | | |
| --- | --- | --- | --- | --- | --- | --- | --- | --- | --- | --- | --- | --- | --- | --- | --- | --- | --- |
|  | aHR | 95% CI | | | *P* |  | aHR | 95% CI | | | *P* |  | aHR | 95% CI | | | *P* |
| 10-year ASCVD risk | 1.01 | 1.00 | - | 1.02 | 0.155 |  | 1.02 | 0.98 | - | 1.06 | 0.314 |  | 1.01 | 0.99 | - | 1.02 | 0.274 |
| Diabetes mellitus | 1.07 | 0.74 | - | 1.55 | 0.701 |  | 0.81 | 0.22 | - | 2.99 | 0.757 |  | 1.09 | 0.74 | - | 1.60 | 0.670 |
| Total PV at baseline | 1.00 | 1.00 | - | 1.00 | <0.001 |  | 1.00 | 1.00 | - | 1.00 | 0.146 |  | 1.00 | 1.00 | - | 1.00 | <0.001 |
| Annualized change of total PV | 1.00 | 1.00 | - | 1.00 | 0.440 |  | 1.00 | 0.98 | - | 1.02 | 0.967 |  | 1.00 | 1.00 | - | 1.01 | 0.507 |
| Statin use | 3.13 | 1.99 | - | 4.94 | <0.001 |  | - | - |  | - | - |  | - | - |  | - | - |
| Cluster 1 (reference) | 1.00 | - |  | - | - |  | 1.00 | - |  | - | - |  | 1.00 | - |  | - | - |
| Cluster 2 | 4.48 | 1.39 | - | 14.45 | 0.011 |  | 1.47 | 0.18 | - | 11.90 | 0.718 |  | 5.89 | 1.42 | - | 24.48 | 0.015 |
| Cluster 3 | 3.55 | 1.11 | - | 11.34 | 0.032 |  | 1.13 | 0.14 | - | 9.09 | 0.911 |  | 4.80 | 1.12 | - | 19.73 | 0.030 |
| Cluster 4 | 3.28 | 1.02 | - | 10.56 | 0.046 |  | 0.78 | 0.09 | - | 7.03 | 0.822 |  | 4.51 | 1.09 | - | 18.65 | 0.038 |
